# COVID-19 Era Effect on Pandemic and Post-pandemic Pediatric Telemedicine Use: A Survey of the European Academy of Pediatrics Research in Ambulatory Settings Network

**DOI:** 10.3389/fped.2021.713930

**Published:** 2021-10-22

**Authors:** Stephen M. Reingold, Adamos Hadjipanayis, Diego van Esso, Stefano del Torso, Hans Jürgen Dornbusch, Ann de Guchtenaere, Rouzha Pancheva, Aida Mujkic, Garyfallia Syridou, Arunas Valiulis, Artur Mazur, Joana Rios, Mateja Vintar Spreitzer, Marina Mamenko, Antonio D'Avino, Gabriela Kubatova, Karin Geitmann, Corrine Wyder, Peter Altorjai, Kyriaki Michailidou, Zachi Grossman

**Affiliations:** ^1^Meuhedet Health Services, Tel Aviv-Yafo, Israel; ^2^Department of Medicine, European University Cyprus, Nicosia, Cyprus; ^3^Centro Médico Pere Grau, Barcelona, Spain; ^4^Pediatria, Childcare Worldwide, Padova, Italy; ^5^Department of Pediatrics, Medical University Graz, Graz, Austria; ^6^Department of Paediatrics, University Hospital Ghent, Ghent, Belgium; ^7^Department of Paediatrics, Regional Hospital AZ Damiaan, Ostend, Belgium; ^8^Department of Hygiene and Epidemiology, Faculty of Public Health, Medical University of Varna, Varna, Bulgaria; ^9^Andrija Štampar School of Public Health, School of Medicine, Univeristy of Zagreb, Zagreb, Croatia; ^10^Department of Pediatrics, School of Medicine, National and Kapodistrian University of Athens, Athens, Greece; ^11^Institute of Clinical Medicine, Medical Faculty, Vilnius University, Vilnius, Lithuania; ^12^Department of Pediatrics and Pediatric Endocrinology and Diabetes, Medical College, University of Rzeszow, Rzeszow, Poland; ^13^Department of Pediatrics, Hospital Beatriz Ângelo, Loures, Portugal; ^14^Health Center Domžale, Domžale, Slovenia; ^15^Department of Pediatrics, Shupyk National Medical Academy of Post-graduate Education, Kyiv, Ukraine; ^16^Pediatria di Famiglia, Federazione Italiana Medici Pediatri FIMP, Naples, Italy; ^17^Paediatric Clinic, Prague, Czechia; ^18^Paediatric Clinic, Hagen, Germany; ^19^Kinderaerzte Kurwerk, Burgdorf, Switzerland; ^20^Paediatric Clinic, Budapest, Hungary; ^21^Biostatistics Unit, Cyprus Institute of Neurology and Genetics, Nicosia, Cyprus; ^22^Adelson School of Medicine, Ariel University, Ariel, Israel; ^23^Maccabi Healthcare Services, Tel Aviv-Yafo, Israel

**Keywords:** pediatrics, telemedicine, COVID-19, tele-pediatrics, hybrid medicine

## Abstract

**Background:** During the COVID-19 pandemic, telemedicine use has increased within community pediatrics. This trend runs counter to reluctance to adaptation of the new mode of healthcare that existed prior to the pandemic. Little is known about what we can expect after the pandemic: if physicians will opt for telemedicine modalities and if tele-pediatrics will continue to be a significant mode of community pediatric care.

**Objective:** The goal of this study was to survey primary pediatric care providers as to their experiences and clinical decision making with telemedicine modalities prior to and during the COVID-19 pandemic, as well as their projected use after the pandemic ends.

**Material and methods:** Using the EAPRASnet database we surveyed pediatricians throughout Europe, using a web-based questionnaire. The survey was performed during the COVID-19 pandemic (June–July 2020), assessed telemedicine use for several modalities, prior to and during the pandemic as well as predicted use after the pandemic will have resolved. Participants were also surveyed regarding clinical decision making in two hypothetical clinical scenarios managed by telemedicine.

**Results:** A total of 710 physicians participated, 76% were pediatricians. The percentage of respondents who reported daily use for at least 50% of all encounters *via* telemedicine modalities increased during the pandemic: phone calls (4% prior to the pandemic to 52% during the pandemic), emails (2–9%), text messages (1–6%), social media (3–11%), cell-phone pictures/video (1–9%), and video conferencing (1–7%) (*p* < 0.005). The predicted post-pandemic use of these modalities partially declined to 19, 4, 3, 6, 9, and 4%, respectively (*p* < 0.005), yet demonstrating a prospectively sustained use of pictures/videos after the pandemic. Reported high likelihood of remotely treating suspected pneumonia and acute otitis media with antibiotics decreased from 8 to 16% during the pandemic to an assumed 2 and 4% after the pandemic, respectively (*p* < 0.005).

**Conclusions:** This study demonstrates an increased utilization of telemedicine by pediatric providers during the COVID-19 pandemic, as well as a partially sustained effect that will promote telemedicine use as part of a hybrid care provision after the pandemic will have resolved.

## Introduction

Telehealth is the delivery of medical care by remote technology. Telemedicine refers particularly to the patient-physician clinical encounter *via* remote technology (1). Prior to 2020, these modalities of medical care have been slowly developing, with limited application and use in community pediatrics (2).

During the COVID-19 pandemic, unprecedented changes were made in the delivery of care in the ambulatory setting. Telehealth has proven its utility, as the use of telemedicine modalities increased during times of social distancing (3). In-person visits to the physician's office declined due to social distancing measures, and as such, telehealth practiced increased. Cautious of in-person interactions, many physicians and patients gravitated to the burgeoning telemedicine options as an alternative to in-office visits (4–6). National guidelines supported this trend, promoting telehealth to mitigate the pandemic while maintaining medical services (7–9).

The European Pediatric Association, Union of National European Pediatric Societies and Associations, demonstrated that pediatric care was sustained during the pandemic through the compensatory use of telemedicine. However, periodic health screening visits and screening programs were significantly reduced (10).

Telemedicine modalities, such as telephone calls, text messages, image or video transfers, video conferencing, or tele-diagnostic devices, are not identical to the traditional “hands-on” approach that physicians were trained to practice. Resistance to change is prevalent among physicians, as is controversy regarding the accuracy of telediagnosis. Previous reports describe overmedication with remote prescribing and raise concern of the deterioration of the physician-patient relationship and the service-oriented nature of medicine that may ensue (11–14).

Prior to the COVID-19 pandemic, telehealth has been promoted globally, at the very least, to provide care to improve accessibility to medical care, and at the most, to ultimately usher in a new model for ideal medical care (15). However, during 2020, telehealth has nevertheless “thrown down the gauntlet,” as it may, challenging today's physicians to accept new technology here and now, in providing primary care.

This change may alter physician's attitudes toward these new modalities to provide medical care and lead to greater utilization of telemedicine modalities in the future (16). Published results of a survey among Israeli physicians demonstrate a limited, albeit significant change in physician's willingness to adapt to telemedicine (17). Previous reports state that clinicians' acceptance of change is the pivotal factor to adaptation (18). The question remains, what lasting effect will the telemedicine experience during this pandemic have on physician behavior and the way care is delivered.

In this study we ascertain physician's impression of telemedicine, inquiring as to their use of telemedicine prior to and during the pandemic, as well as to what extent they expect telemedicine to remain part of their medical practice in the future, after the pandemic.

## Methods

### Study Sample

During the early phases of the pandemic, we utilized the EAPRAS network to conduct a survey of primary care pediatric care providers throughout the continent. The European Academy of Pediatrics Research in Ambulatory Settings Network (EAPRASnet), established in 2009, is a practice-based research network of primary care pediatricians affiliated with the European Academy of Pediatrics. The network has previously been involved in studies performed in primary care (19–22).

### Data Collection

Data were collected using a web-based questionnaire that was posted on the home page of the EAPRASnet during June-July 2020. Only registered physicians were permitted to participate. Respondents were invited to participate *via* email using a mailing list that had been created for previous EAPRASnet projects. Pediatric care providers not belonging to the network were invited by national EAPRASnet coordinators to complete the questionnaire. Three reminders were sent out *via* email, and data were collected till the end of July 2020.

### Socio-Demographic Details

Participants were asked for their gender, age, years in practice, medical specialty, and place of work.

### Use of Telemedicine Modalities

The first part of the survey inquired about frequency of use of various telemedicine modalities prior to and during the epidemic, as well-expected use after the epidemic. Participants were asked specifically regarding phone calls, text messages, photo/video, email, and video conferencing.

### Clinical Scenarios

The latter part of the survey consisted of two hypothetical scenarios and evaluated the decision to manage remotely.

Case 1, a suspected pneumonia, was presented as:

“*The parents of a 7-year-old girl contact you and report that the child has had 4 days of high fever, cough, and nasal congestion. The child is not in distress, has mild anorexia, no vomiting and passed two loose stools today.”*

Respondents were asked to rate the likelihood of making an empiric diagnosis, prescribing antibiotic treatment, prescribing symptomatic treatment, referring for a chest X-ray, referring to an emergency room, and in the case of wheezing, prescribing corticosteroids.

Case 2, a suspected otitis media, was presented as:

“*The parents of a 2-year-old boy contact you and report that the child has had fever for 2 days, mild upper respiratory symptoms and left ear pain. The child is vigorous, eating well, had one loose stool, but slept poorly last night.”*

Respondents were asked to rate the likelihood of making an empiric diagnosis, prescribing antibiotic treatment, and prescribing symptomatic treatment.

In both scenarios, respondents were to assume that the patient is known to the respondent and that there is no suspicion of COVID-19 for the case. Participants were asked to answer each question as if they would have practiced prior to the pandemic, currently practice during the pandemic and how they foresee themselves practicing after the pandemic. Responses were evaluated using a 5-point Likert scale ranging from “certainly not” to “certainly will.”

### Data Analysis

Demographic data were presented as percentages of the total responses. Evaluation of the answers to the use of telemedicine technologies and responses to the two clinical scenarios between pre-, during and post-COVID-19 periods were compared using chi-square tests. Statistical significance was considered if the *p*-value was <0.05. Analyses were performed using R statistical software. The raw data is available in the Supplementary Material.

## Results

### Demographic Data

**Figure 1:**
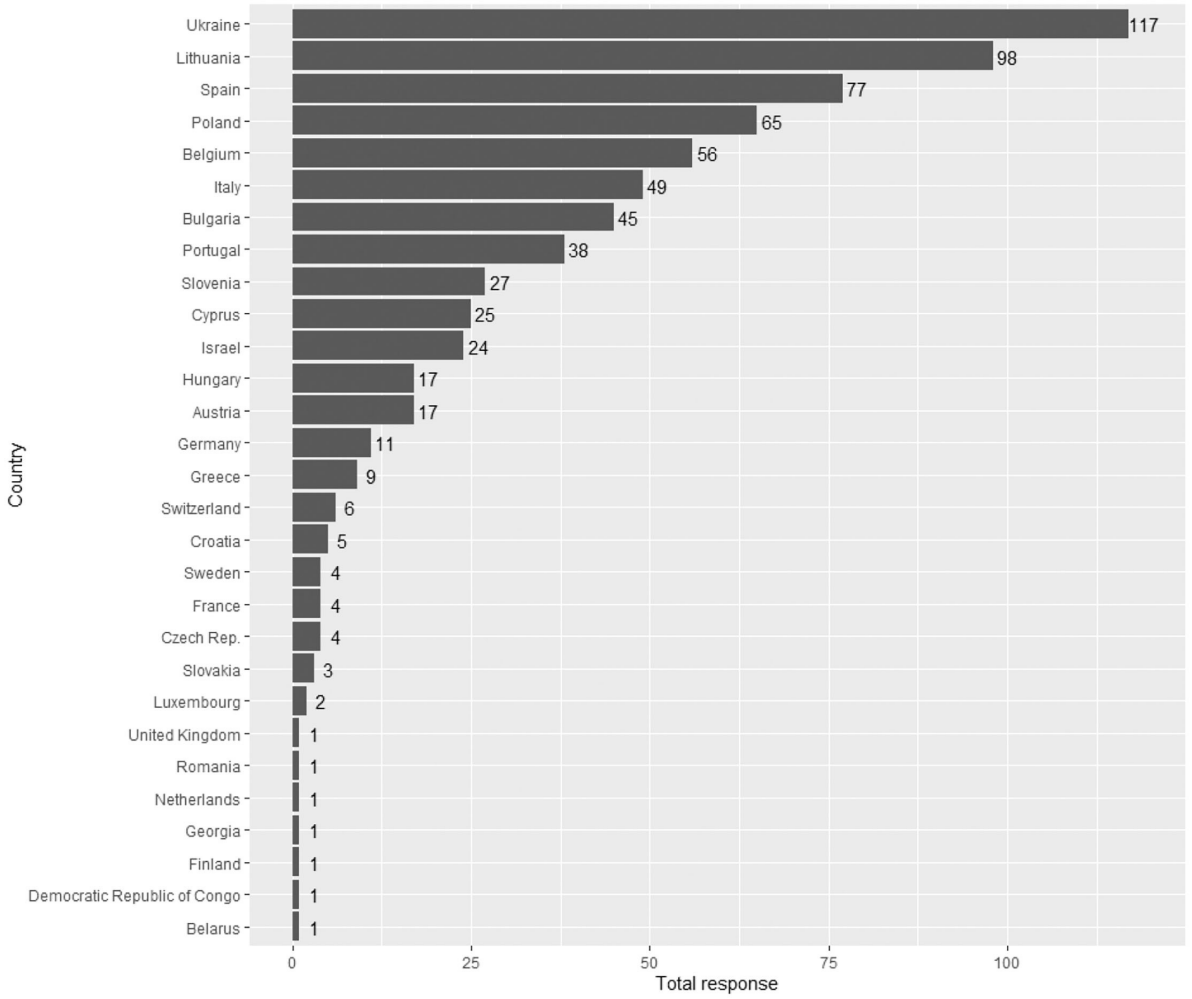
Distribution of participants per country.

A total of 710 physicians responded to the survey, 77% were female, 76% were pediatricians, and 17% were family or general practitioners. Details regarding age, specialty and years of experience are presented on Table 1. Geographic distribution of respondents is presented on Figure 1.

**Table 1:** Demographic characteristics of participant physicians.

		***N* = 710**	***%***
**Age**			
	≤40	198	28
	41–50	141	20
	51–60	218	31
	61–70	136	19
	>70	17	2.4
**Gender**			
	Male	165	23
	Female	545	77
**Years of experience**			
	≤10	166	23
	11–20	146	21
	21–30	206	29
	>30	192	27
**Specialty**			
	Pediatrics	537	76
	Family practice/GP	123	17
	Internal medicine	6	0.8
	Other	44	6.2

### Use of Telemedicine Modalities

Table 2 represents the use of telemedicine modalities for at least 50% of all daily encounters, including phone calls, emails, text messages, social media, cell-phone picture/video, and video conferencing. Usage is reported for prior to the pandemic and during the pandemic, as well as projected use after the pandemic.

**Table 2:** Use of telemedicine modalities for at least 50% of daily encounters.

**Modality**	**Respondents** ***N*** **=** **710**					
	**Before COVID-19**		**During COVID-19**		**After COVID-19**	
	***N***	***%***	***N***	***%***	***N***	***%***
Phone calls	28	4	371	52^*^^,^^+^	135	19^#^
Emails	11	2	65	9^*^^,^^+^	31	4^#^
Text messages	8	1	46	6^*^^,^^+^	19	3^#^
Social media	21	3	78	11^*^^,^^+^	46	6^#^
Cell-phone pictures/video	10	1	64	9^*^	64	9^#^
Video conference	7	1	53	7^*^^,^^+^	25	4^#^

* *During vs. before, p < 0.05*.

+ *During vs. after, p < 0.05*.

# *After vs. before, p < 0.05*.

Reported use of all telemedicine modalities increased significantly during the pandemic. For all modalities except for pictures/video, the degree of use is predicted to decrease after the pandemic yet remain significantly greater than prior to the pandemic. Post-pandemic predicted picture/video use is not only higher than pre-pandemic use but is predicted to remain at the same level as pandemic era use.

### Scenario 1

Table 3 demonstrates clinical decision-making patterns for Scenario 1, the high-likelihood (likely to or certainly will) to manage remotely, and more specifically, to provide antibiotics, provide symptomatic care, refer for chest X-ray, refer to the ED, or prescribe steroids, all without having seen the patient in-person. All management options *via* telemedicine increased in frequency during the pandemic and yet all are predicted to reduce in frequency after the pandemic. The option to manage remotely is predicted to return to near pre-pandemic frequency, as are most management options. Only referrals to X-ray and the ED are predicted to have any increase in frequency sustained after the pandemic, when compared to pre-pandemic levels.

**Table 3:** High likelihood pattern of clinical decision making in scenario 1.

**Clinical decision making**	**Respondents** ***N*** **=** **710**					
	**Before COVID-19**		**During COVID-19**		**After COVID-19**	
	***N***	***%***	***N***	***%***	***N***	***%***
Manage remotely	130	18	258	36^*^^,^^+^	159	22
Provide antibiotics without examination	10	1.4	56	8^*^^,^^+^	16	2
Provide symptomatic care without examination	172	24	320	45^*^^,^^+^	205	29
Ask for an X-ray without examination	11	1.6	70	10^*^^,^^+^	30	4.2^#^
Refer to ED without examination	34	4.8	164	23^*^^,^^+^	61	8.6^#^
Prescribe steroids without examination	62	8.7	169	24^*^^,^^+^	84	12

* *During vs. before, p < 0.05*.

+ *During vs. after, p < 0.05*.

# *After vs. before, p < 0.05*.

### Scenario 2

Table 4 demonstrates clinical decision-making patterns for Scenario 2, the high-likelihood (likely to or certainly will) to manage remotely, and more specifically, to provide antibiotics or symptomatic care without an in-person visit. Managing and treating *via* telemedicine increased significantly during the pandemic when compared to the pre-pandemic rates. Predicted use of telemedicine to manage and provide symptomatic care after the pandemic dropped significantly; however, when compared to pre-pandemic patterns, a significant increase in frequency is still sustained. Yet, post-pandemic antibiotic prescribing *via* telemedicine is predicted to return to near pre-pandemic levels, with no significant change predicted for the time after the pandemic, when compared to the pre-pandemic period.

**Table 4:** High likelihood pattern of clinical decision making in scenario 2.

**Clinical decision making**	**Respondents** ***N*** **=** **710**					
	**Before COVID-19**		**During COVID-19**		**After COVID-19**	
	***N***	***%***	***N***	***%***	***N***	***%***
Manage remotely	71	10	284	40^*^^,^^+^	119	17^#^
Provide antibiotics without examination	17	2	113	16^*^^,^^+^	31	4
Provide symptomatic care without examination	220	31	388	55^*^^,^^+^	144	20^#^

* *During vs. before, p < 0.05*.

+ *During vs. after, p < 0.05*.

# *After vs. before, p < 0.05*.

## Discussion

Our study demonstrates increased utilization of telemedicine for outpatient pediatric care during the COVID-19 pandemic. This increase was reported for all modalities considered: telephone calls, text messages, photo/video clips, and video calls, both when considering overall use, as well as when applied to specific clinical scenarios. These findings are consistent with other studies demonstrating physician's willingness to quickly adapt to telemedicine practices during the pandemic (3–6). However, for the first time, according to our knowledge, we could demonstrate that the use of all telemedicine modalities was predicted to remain in increased use after the pandemic, when compared to pre-pandemic levels. Experience during the pandemic has brought primary pediatric care to the forefront of telemedicine use.

A similar survey was previously used in a study of Israeli participants demonstrating a similar adaptation to telemedicine during the pandemic, yet it could not demonstrate similar willingness to adopt such practices after the pandemic (17). The difference between the studies may be the result of population variances. Additionally, the smaller sample size of the Israeli study (170 pediatricians) may not have had the power to detect significant changes for the post-pandemic phase. However, there may be more at hand. This European study was performed in June and July 2020, immediately after the completion of the Israeli study (May 2020). Though only 2 months following, we surmise that as physicians continued to use telemedicine, they became more comfortable with the modality thereby increasing their willingness to use it under “normal” circumstances.

As medical care advances during the twenty-first century, a hybrid of physician-patient interactions is developing, consisting of both in-person and telemedicine visits. However, whereas previous goals may have been created to utilize telemedicine to access hard to reach patients, crossing distances or physical barriers, current COVID-19 era use has forged a new ideal. Current telemedicine use has demonstrated that a significant percentage of physician-patient encounters does not require an in-person visit. The convenience of interacting remotely may increase efficiency for certain aspects of primary pediatric care.

Prior to the COVID-19 pandemic, strides in telemedicine were lagging for community pediatrics. Previous studies demonstrate physician's resistance to telemedicine utilization for reasons that include: lack of reimbursement, lack of infrastructure, non-familiarity with new modalities, discomfort with adopting new technologies, lack of confidence in the accuracy of tele-diagnosis, ease of missing or delaying critical diagnoses (such as medical emergencies or cancer), predisposition to ancillary testing and antibiotic prescribing with telemedicine use, lack of human interaction and impaired interpersonal dialogue that is critical to the physician-patient relationship (23–26).

The current COVID-19 pandemic has forced the primary care pediatrician's hand in utilization of telemedicine for a lack of a better alternative. We predict that this recent use will engender favorable attitudes toward the modality and lead to sustained use after the pandemic resolves.

We suggest that increased use during this era has increased familiarity, reduced “techno-phobia,” allowed for the learning of meaningful use, as well as demonstrated telemedicine's opportunities for efficiency and convenience. Experience during the pandemic allows for the development of meaningful use both on the individual and on the institutional level with the development of protocols and guidelines (27, 28).

Clinical scenarios that require the elucidation of physical findings for diagnostic accuracy pose a unique challenge to telemedicine. In the clinical scenarios we presented, physicians were willing to diagnose and prescribe empirically and without a physical exam only during the pandemic era and expect to return to in-patient visits after the pandemic. However, as new technologies offer “telediagnostics,” whereby part of the physical exam may be performed remotely, expectations are likely to change. New devices allow for auscultation, otoscopy, and visualization of the oropharynx *via* a remote device. Such devices have recently proven to provide high quality sound and images, on par with traditional medical devices (29, 30).

External physical findings, such as dermatologic exams have demonstrated increased utility for telemedicine use. Of all modalities surveyed in our study, images/video clips is the only modality to have predicted future use to remain sustained at pandemic levels. Advanced cell-phone camera technology has made it easy for taking and sending quality photographs. Studies have demonstrated that most skin lesions in pediatric primary care attention could be managed by tele-dermatology (31, 32).

However, increased utilization of telemedicine during COVID-19 for the clinical scenarios presented elucidate a particular need for caution with telemedicine: over-prescription. Previous studies have shown an increased frequency in antibiotic prescribing *via* telemedicine as compared to in-person visits (26). This tendency was confirmed by our presented case scenarios, with an increase in tele-prescribing during the pandemic. One may counter-argue, however, that our study of pandemic era practices presents a unique phenomenon during necessary social distancing, one that would not necessarily hold true during “normal” circumstances.

Our study has several limitations. Subjective and predictive reporting of use does not reflect the same accuracy as objective measures. Those who responded may not actually represent the opinions of all physicians in pediatric primary care. Participation was not proportional across all countries, and this may alter the study's ability to represent all of Europe. However, the limitation of the sample representativeness should be considered in the context of a first study of its kind, bringing data originating from primary care pediatricians. The pandemic spread varied in different countries, which may have influenced survey results. Future use are predictions made in the earlier part of the pandemic. Predictions may not be entirely accurate as attitudes may change as the pandemic persists and use of telemedicine (now for almost 2 years) is prolonged.

Our study did not explore chronic care, preventive care, or behavioral and developmental pediatrics. Unlike the clinical acute care scenarios presented, chronic care, preventive care and behavioral and developmental management may have greater utility for telemedicine (33, 34).

Our study did not explore the “other side of the examination table,” or how patients feel about telemedicine. Initial studies in pediatric telemedicine are optimistic, demonstrating significant patient satisfaction (35). This may drive pressure on physicians to provide a service that patients demand. Health maintenance organizations and other healthcare organizations may require such practice from their providers to save costs, improve quality of care, increase patient compliance or simply as a means to attract patients. The ethics of utilization must also be explored.

We suggest further studies that (1) assess *via* objective measures (2), differentiate between visits that focus more on a physical exam vs. those that relate to preventive, behavioral, developmental, or administrative issues, as well as chronic care (3), assess the use of pediatric telediagnostics, and (4) reassess over time.

Additional studies will be needed to assess the cost of telemedicine as well as physician and patient satisfaction with its use. Ethical use, the quality of its care and liability issues must be further explored, so that evidence-based guidelines and protocols can be developed to guide further telemedicine use (36, 37).

In conclusion, our study demonstrates an increased use of telemedicine in primary care pediatrics during the COVID-19 epidemic, as well as its predicted effect of greater telemedicine use in the future after the pandemic, compared to the pre-pandemic situation.

## Data Availability Statement

The raw data supporting the conclusions of this article will be made available by the authors, without undue reservation.

## Author Contributions

SR and ZG conceived of and designed the study and composed the manuscript. KM performed statistical analysis. AH, DE, ST, and HD provided analytical and editorial review. AG, RP, AMu, GS, AV, AMa, JR, MS, MM, AD'A, GK, KG, CW, and PA coordinated data collection. All authors have discussed the results and contributed to the final manuscript.

## Conflict of Interest

SR is a paid consultant of Tytocare. The remaining authors declare that the research was conducted in the absence of any commercial or financial relationships that could be construed as a potential conflict of interest.

## Publisher's Note

All claims expressed in this article are solely those of the authors and do not necessarily represent those of their affiliated organizations, or those of the publisher, the editors and the reviewers. Any product that may be evaluated in this article, or claim that may be made by its manufacturer, is not guaranteed or endorsed by the publisher.
